# Altered Gut Microbial Profile Accompanied by Abnormal Fatty Acid Metabolism Activity Exacerbates Endometrial Cancer Progression

**DOI:** 10.1128/spectrum.02612-22

**Published:** 2022-10-13

**Authors:** Shan-Shan Zhao, Lei Chen, Jing Yang, Zhen-Hua Wu, Xiao-Yu Wang, Qi Zhang, Wen-Jie Liu, Hui-Xin Liu

**Affiliations:** a Health Sciences Institute, China Medical University, Shenyang, Liaoning Province, People’s Republic of China; b School of Life Sciences, China Medical University, Shenyang, Liaoning Province, People’s Republic of China; c Liaoning Key Laboratory of Obesity and Glucose/Lipid Associated Metabolic Diseases, China Medical University, Shenyang, Liaoning Province, People’s Republic of China; d Department of Gynecology, Cancer Hospital of China Medical University, Liaoning Cancer Hospital & Institute, Shenyang, Liaoning Province, People’s Republic of China; Shandong University

**Keywords:** microorganisms, host-microbe relationships, dysbiosis, obesity, tumor microenvironment, metabolic syndrome, biomarker

## Abstract

Endometrial cancer (EC) is the most prevalent gynecological malignancy, with a higher risk in obese woman, indicating the possibility of gut microbiota involvement in EC progression. However, no direct evidence of a relationship between EC and gut microbiota in humans has been discovered. Here, we performed 16S rRNA sequencing to explore the relationship between dysbiosis of gut microbiota and cancer development in different types of EC patients. The results clearly show the differential profiles of gut microbiota between EC patients and normal participants as well as the association between gut microbiota and EC progression. Targeted metabolomics of plasma revealed an increased level of C16:1 and C20:2, which was positively associated with the abundance of *Ruminococcus* sp. N15.MGS-57. The higher richness of *Ruminococcus* sp. N15.MGS-57 in EC subjects not only was positively associated with blood C16:1 and C20:2 but also was negatively correlated with betalain and indole alkaloid biosynthesis. Furthermore, the combined marker panel of gut bacteria, blood metabolites, and clinical indices could distinguish the EC patients under lean and overweight conditions from normal subjects with high accuracy in both discovery and validation sets. In addition, the alteration of tumor microenvironment metabolism of EC was characterized by imaging mass microscopy. Spatial visualization of fatty acids showed that C16:1 and C18:1 obviously accumulate in tumor tissue, and C16:1 may promote EC cell invasion and metastasis through mTOR signaling. The aberrant fecal microbiome, more specifically, *Ruminococcus* sp. N15.MGS-57 and spatially distributed C16:1 in EC tissues, can be used as a biomarker of clinical features and outcomes and provide a new therapeutic target for clinical treatment.

**IMPORTANCE** A growing number of studies have shown the connection between gut microbiota, obesity, and cancer. However, to our knowledge, the association between gut microbiota and endometrial cancer progression in humans has not been studied. We recruited EC and control individuals as research participants and further subgrouped subjects by body mass index to examine the association between gut microbiota, metabolites, and clinical indices. The higher richness of *Ruminococcus* sp. N15.MGS-57 in EC subjects was not only positively associated with blood C16:1 but also negatively correlated with betalain and indole alkaloid biosynthesis. Spatial visualization of fatty acids by imaging mass microscopy showed that C16:1 obviously accumulates in tumor tissue, and C16:1 may promote the EC cell invasion and metastasis through mTOR signaling. The aberrant fecal microbiome, more specifically, *Ruminococcus* sp. N15.MGS-57 and spatially distributed C16:1, can be used as a biomarker of clinical features and outcomes and provide a new therapeutic target for clinical treatment.

## INTRODUCTION

Endometrial cancer (EC) is the most prevalent gynecological malignancy, with increasing incidence rates accompanied by accelerated increasing mortality in some developed countries (1). Traditionally, EC has been classified into two histological categories, type I and type II. Type I is mostly associated with obesity and other components of the metabolic syndrome, which has been identified as an independent risk factor for the development of EC. Type II tumors behave more aggressively and are not estrogen driven. It has been known that type II EC is not completely devoid of associations with hormonal and metabolic factors. However, recent epidemiological studies indicate a complexity in EC risk factors, with obesity playing an important role in both types (2). The incidence of obesity has increased dramatically. Sedentary lifestyles and increased food consumption in combination with a widespread polygenetic susceptibility are the major causes of the obesity epidemic. Microorganisms have developed intimate relationships with humans by colonizing various body environments, constituting an integrated metaorganism. As a nonnegligible body component, microbes may directly or indirectly modulate cancer susceptibility and tumor progression (3, 4). The human microflora contributes to 16 to 18% or more of worldwide malignancies. Oncogenic bacteria and viruses exhibit the capacity to directly modulate carcinogenesis through specific toxins that can damage host DNA or the integration of oncogenes into host genomes. Gut microbial dysbiosis has been related to cancer based on current epidemiological and experimental evidence (5). Commensal bacteria can modulate host homeostatic processes, and the specific subsets of microorganisms directly influence the human physiology through their metabolites (6). Microbially driven carcinogenesis is also frequently related to global changes in the microbiome (7). Helicobacter pylori, which is known for its association with both lymphoma and gastric epithelial cancer, appears to use many parallel mechanisms to induce cancer (8). *Fusobacterium* causes inflammation, proliferation, and loss of immune surveillance. Multiple laboratories have observed increases in *Fusobacterium* species in colorectal cancer samples (9). However, the composition of the gut microbiota in patients with EC and the relationship between the gut microbiota and EC have not been clarified so far.

Metabolic alteration is a characteristic of malignancy that was first recognized a century ago. Many cancer-specific metabolic alterations have been described, including the aberrant metabolism of amino acids, glucose, nucleotides, fatty acids, and lipids. Alterations in intracellular and extracellular metabolites that can accompany cancer-associated metabolic reprogramming have profound effects on gene expression, cellular differentiation, and the tumor microenvironment (10). Fatty acids determine the enormous structural complexity. Moreover, fatty acids are energy-rich compounds that can be degraded to provide ATP and contribute to cellular bioenergetics. The regulation of fatty acid synthesis, modification, uptake, and degradation is therefore essential for the maintenance of cellular physiology, and perturbation of the processes controlling lipid provision can inhibit cell survival. Altered fatty acid metabolism is among the most prominent metabolic alterations in cancer. Studies highlight the relationship between oncogenic signaling and fatty acid metabolism to promote cancer cell growth and survival, to regulate the processes that initiate cell dissemination and metastasis, and to control communication between cancer and immune cells (11).

Mounting evidence has documented the connection between the gut microbiota, estrogen metabolism, and obesity, which suggest a potential role of the microbiome in the etiology of EC (12). The gut microbiota can produce various bioactive metabolites, which can enter the bloodstream of the host through absorption into the enterohepatic circulation. Specific metabolites associated with a disease phenotype can be identified by mass spectrometry or nuclear magnetic resonance-based metabolomics of fecal, plasma, urine, or other biofluids, making it possible to conduct joint analyses of the microbiome, metabolome, and host phenotypes to identify potential mechanistic links (13). In this study, we analyzed and compared the composition and functional potential of the gut microbiome in EC patients with those in healthy controls, utilizing 16S rRNA sequencing. By combining metabolomics, we aimed to further explore the gut microbiota and metabolome as being altered in association with EC and to determine whether they play a considerable role in the initiation and progression of this disease. Obesity, endometrial intraepithelial neoplasia and hypertension as high-risk factors were also analyzed in a subgroup.

## RESULTS

A total of 18 EC patients and 18 control (normal) participants took part in the study (normal participants included those in the normal weight control [NC, *n* = 6] group, those in the normal overweight [NO, *n* = 6] group, and those in the endometrial intraepithelial neoplasia with overweight [EIN, *n* = 6] group; EC patients included those in the EC normal weight control [ECC, *n* = 6] group, those in the EC with overweight [ECO, *n* = 6] group, and those in the EC with overweight and hypertension [ECOH, *n* = 6] group). We used body mass index (BMI) for grouping. According to the World Health Organization (WHO) criteria, overweight is defined as a BMI greater than or equal to 25 kg/m^2^ and a normal weight is defined as a BMI less than 25 kg/m^2^. The baseline characteristics of the EC group and the control group are summarized in Table S1 in the supplemental material.

### Alterations of gut microbiota composition in normal and EC patients based on 16s rRNA sequencing.

**FIG 1:**
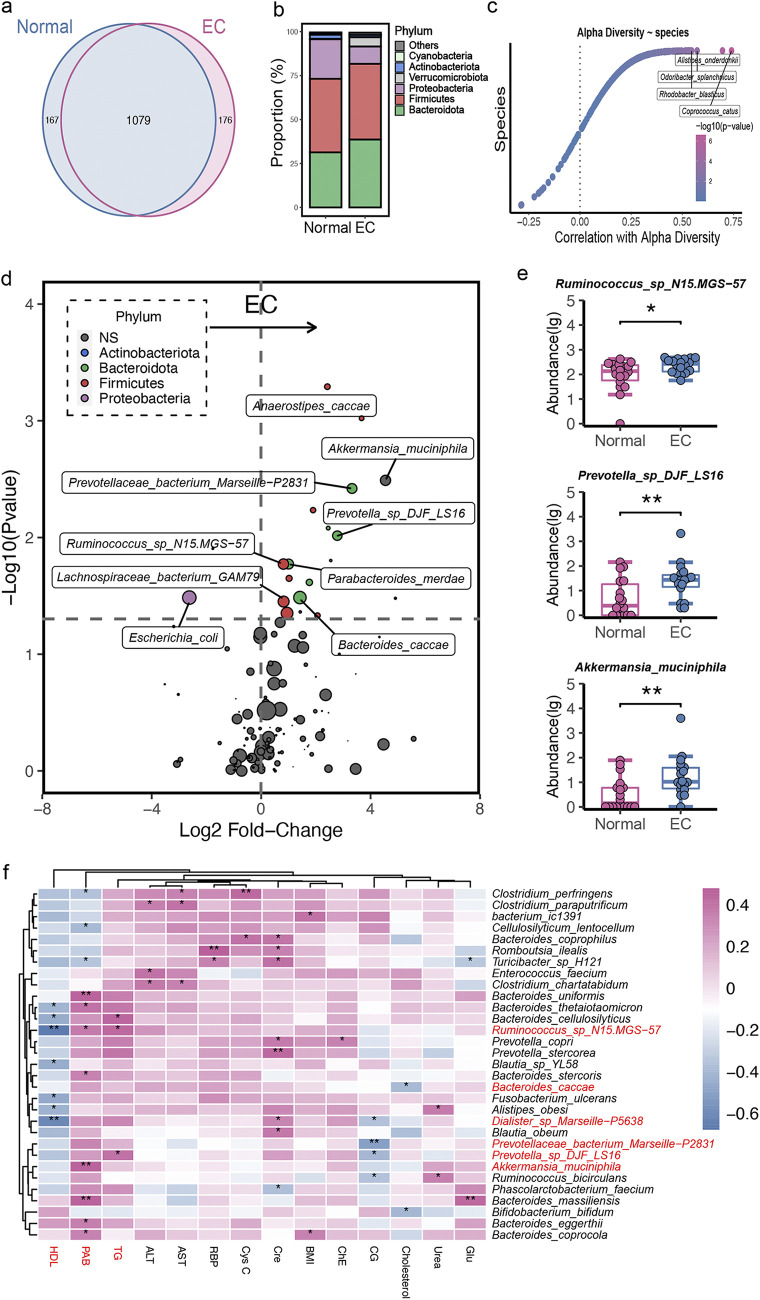
Gut microbial alterations in normal and EC individuals. (a) Venn diagram of the observed OTUs in normal and EC groups. (b) Bacterial profile at the phylum level. (c) Correlation between alpha-diversity and gut bacteria. (d) Volcano plots showing the changes in gut bacteria between normal and EC subjects at the species level. Significantly different taxa are colored according to the phyla as indicated in the key. The size of the plots indicates the abundance of the gut bacteria. (e) The absolute abundance of species in the indicated groups (a two-tailed Wilcoxon test was used to determine significance in normal and EC individuals; *, *P < *0.05; **, *P < *0.01). (f) Spearman’s rank correlation between the top 30 species and 14 clinical indices (only species that correlated with at least one clinical index at a *P *of <0.05 [*] or a *P *of <0.01 [**] are shown).

As shown in Fig. 1a, a Venn diagram displayed 1,079 common operational taxonomic units (OTUs) with 176 unique OTUs in the EC group and 167 unique OTUs in the normal group. The sequencing results showed no significant difference in bacterial alpha-diversity and beta-diversity between individuals with EC and normal participants (Fig. S1a and S1b). At the phylum level (Fig. 1b), the abundance of *Proteobacteria* was decreased in the EC group, while *Bacteroidota* and *Verrucomicrobiota* were increased in the EC group. Several microbial species, such as Coprococcus catus, Rhodobacter blasticus, Odoribacter splanchnicus, and Alistipes onderdonkii, were strongly correlated with microbial diversity, which indicates a potential role for these discriminatory species in maintaining microbiome richness (Fig. 1c). Furthermore, the bacteria belonging to the phyla *Firmicutes* and *Bacteroidota* dominated in EC subjects, with higher proportions (Fig. 1d). Specifically, *Firmicutes* phylum members *Ruminococcus* sp. N15.MGS-57, *Lachnospiraceae* bacterium GAM79, and Anaerostipes caccae were enriched in EC patients. *Bacteroidota* phylum members Alistipes indistinctus, Parabacteroides merdae, *Prevotellaceae* bacterium Marseille-P2831, and *Prevotella* sp. DJF_LS16 species were higher in proportion in EC subjects. In addition, Akkermansia muciniphila was also higher in EC subjects (Fig. 1e).

To further explore potential correlations of key clinical indexes with the altered gut microbiome in EC, clinical index and gut microbiome correlation analysis was performed. We found that BMI and 13 metabolic parameters, including serum high-density lipoprotein (HDL) and alanine aminotransferase (ALT), correlated with gut bacterial alterations (Fig. 1f). Specifically, *Ruminococcus* sp. N15.MGS-57 and *Dialister* sp. Marseille-P5638 were negatively correlated with circulating HDL, which was mainly produced by the liver and responsible for cholesterol transport, and were positively correlated with circulating triglycerides (TG) and creatinine (Cre) concentration, respectively. Moreover, Akkermansia muciniphila, which also increased in EC individuals, was positively associated with the serum prealbumin (PAB). Furthermore, Bacteroides coprocola was positively correlated with BMI, indicating that these species disturbances may constitute potential biomarkers linking gut microbiota and metabolic status. Besides, the species cooccurrence network revealed that there was a closer correlation among Prevotella copri, *Parabacteroides* sp. CT06, and related species in EC subjects (Fig. S2a). Normal and EC groups shared only a small proportion of edges (9 edges) but had dramatically different numbers of unique edges (44 versus 31) (Fig. S2b). The eigenvectors of shared nodes between the normal and EC groups were also quite different (Fig. S2c). Moreover, the random forest model was used to discriminate individuals with EC from normal participants based on the species level. The optimal model utilized 9 species, which provided the lowest cross-validation error (Fig. S2d and e) and a high discriminatory power with the area under the curve (AUC = 0.74) in the validation set (Fig. S2f).

### Characteristics of metabolic symbols of EC patients.

**FIG 2:**
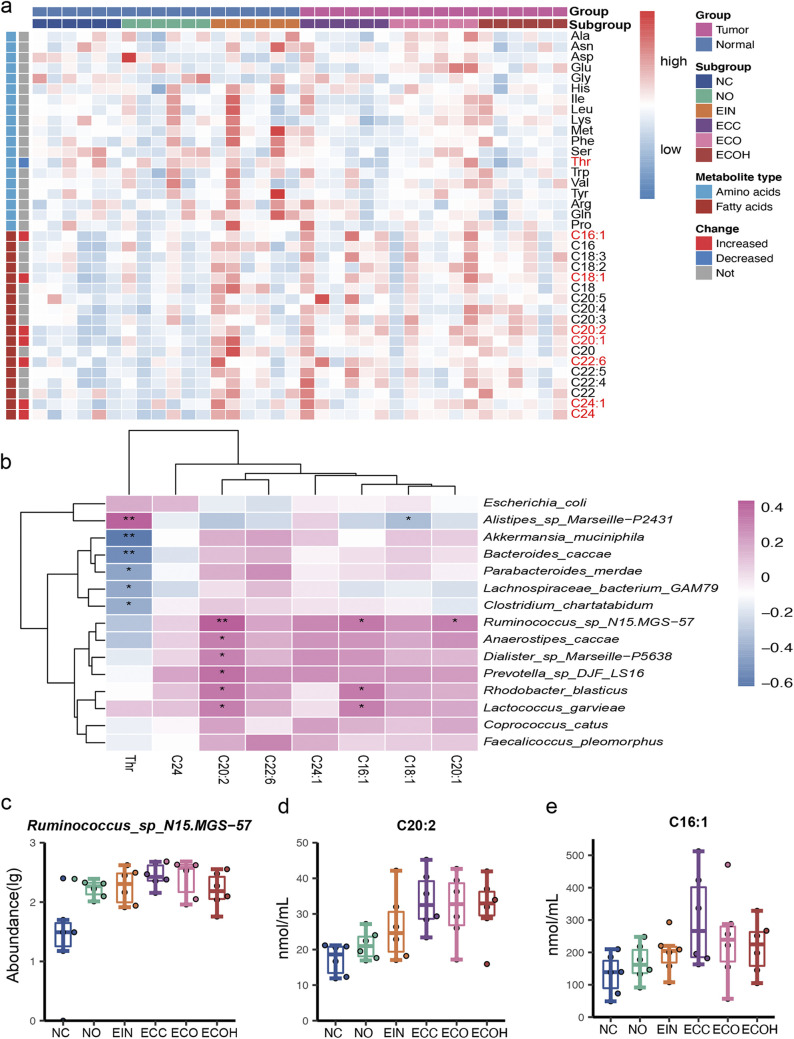
Targeted metabolomics reveals the host circulating metabolite characteristics. (a) Heat map displaying the metabolite signal intensity across individuals. (b) Correlation of the significant change in metabolites and gut bacteria. (c to e) Abundance of the gut bacterium *Ruminococcus* sp. N15.MGS-57 and related metabolites C20:2 and C16:1 in 6 groups.

To investigate the extent to which circulating metabolites were associated with the altered microbiome in the EC patients, targeted metabolomics profiling of serum was performed in EC patients and controls. We identified 8 metabolites that significantly differed in abundance between two groups. Specifically, C16:1, C18:1, C20:1, C20:2, C22:6, C24, and C24:1 were enriched in EC subjects, while a decreased level of threonine was observed in EC patients (Fig. 2a). Moreover, Spearman’s correlation was generated to explore the potential relationships between the gut microbiome changes and the altered abundance of circulating metabolites. As shown in Fig. 2b, EC enriched metabolites C16:1 and C20:2 were positively associated with *Ruminococcus* sp. N15.MGS-57, *Prevotella* sp. DJF_LS16, and Anaerostipes caccae, which dominated in EC patients. Apart from that, Akkermansia muciniphila, Bacteroides caccae, Parabacteroides merdae, and *Lachnospiraceae bacterium* GAM79 bacteria were negatively associated with the circulating threonine, which dominated in normal subjects. More importantly, *Ruminococcus* sp. N15.MGS-57 bacteria and metabolic products C16:1 and C20:2 demonstrated similar rising trends in abundance from normal subjects to EC patients (Fig. 2c to e).

### Gut microbiota composition in normal weight controls and EC normal weight controls.

**FIG 3:**
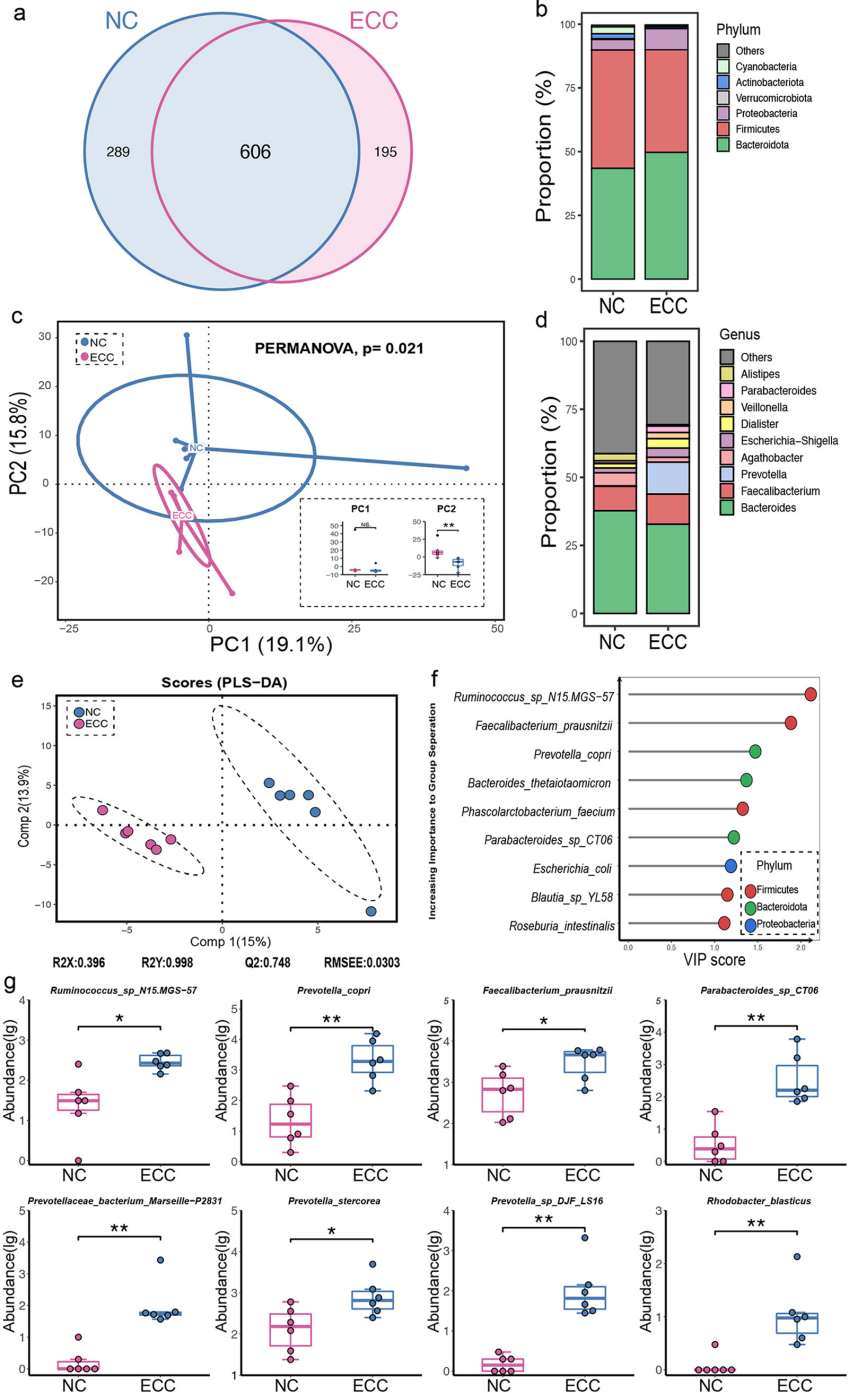
Construction and biomarker species in EC patients of normal weight. (a) Venn diagram of the observed OTUs in NC and ECC individuals. (b) Comparison of the microbiota profiles of NC and ECC groups at the phylum level. (c) Beta-diversity between two groups. (d) Comparison of the microbiota profiles of NC and ECC groups at the genus level. (e) Clustering analyses of PLS-DA score plot of species abundance in samples from individuals from NC (blue points) and ECC (red points) groups. (f).VIP scores of PLS-DA. VIP scores were used to rank the discriminating powers of different taxa between the NC and ECC groups (VIP score of >1). (g) Abundance of significantly changed species in NC and ECC individuals (two-tailed Wilcoxon test; *, *P < *0.05; **, *P < *0.01).

As shown in Fig. 3a, the unique numbers of OTUs in the NC and ECC groups present a significant difference. At the phylum level, the proportions of *Bacteroidota* and *Proteobacteria* were increased in the ECC group but a reduction in *Firmicutes*, *Actinobacteriota*, and *Cyanobacteria* was observed in ECC subjects (Fig. 3b). To explore whether the microbial composition of ECC subjects was different from that of NC subjects, alpha-diversity analysis was performed. Even though there was no significant difference across the two groups in the alpha-diversity indexes (Fig. S1c), overall microbial composition was significantly different between the ECC and NC groups, as confirmed by the permutational multivariate analysis of variance (PERMANOVA) test (*P = *0.021) (Fig. 3c). This difference arose from principal component 2 (PC2) (**, *P* < 0.01) rather than PC1 (*P* > 0.05) of the principal-component analysis (PCA). Notably, the genera *Prevotella* and *Parabacteroides* showed a remarkable increase in the ECC group (Fig. 3d). Partial least-squares discriminant analysis (PLS-DA) showed that there was a distinct clustering pattern between samples from individuals in the ECC and NC groups (Fig. 3e). Also, the variable importance in projection (VIP) score for the gut microbiota showed that *Ruminococcus* sp. N15.MGS-57 contributed significantly to the group separation (Fig. 3f). Apart from that, the species *Ruminococcus* sp. N15.MGS-57, Faecalibacterium prausnitzii, Prevotella copri, and *Parabacteroides* sp. CT06, which belong to the *Firmicutes* and *Bacteroidota* phyla, were significantly increased in ECC individuals compared to the NC group (Fig. 3g).

In addition, representative sequences of each OTU were used to predict metabolic pathways. As shown in Fig. S3a, pathways involved in the renin-angiotensin system and in betalain and indole alkaloid biosynthesis were present at a significantly lower level in ECC individuals than in the NC group. And the correlation between altered species and KEGG pathways revealed that *Parabacteroides* sp. CT06, Prevotella copri, and *Ruminococcus* sp. N15.MGS-57, which increased in ECC patients, were negatively associated with the above-described pathway (Fig. S3b). Furthermore, a positive association between altered bacteria and increased circulation lipids such as C16:1 and C20:2 was also observed in lean subjects (Fig. S3c).

### Changes in gut microbiota profile due to overweight condition.

**FIG 4:**
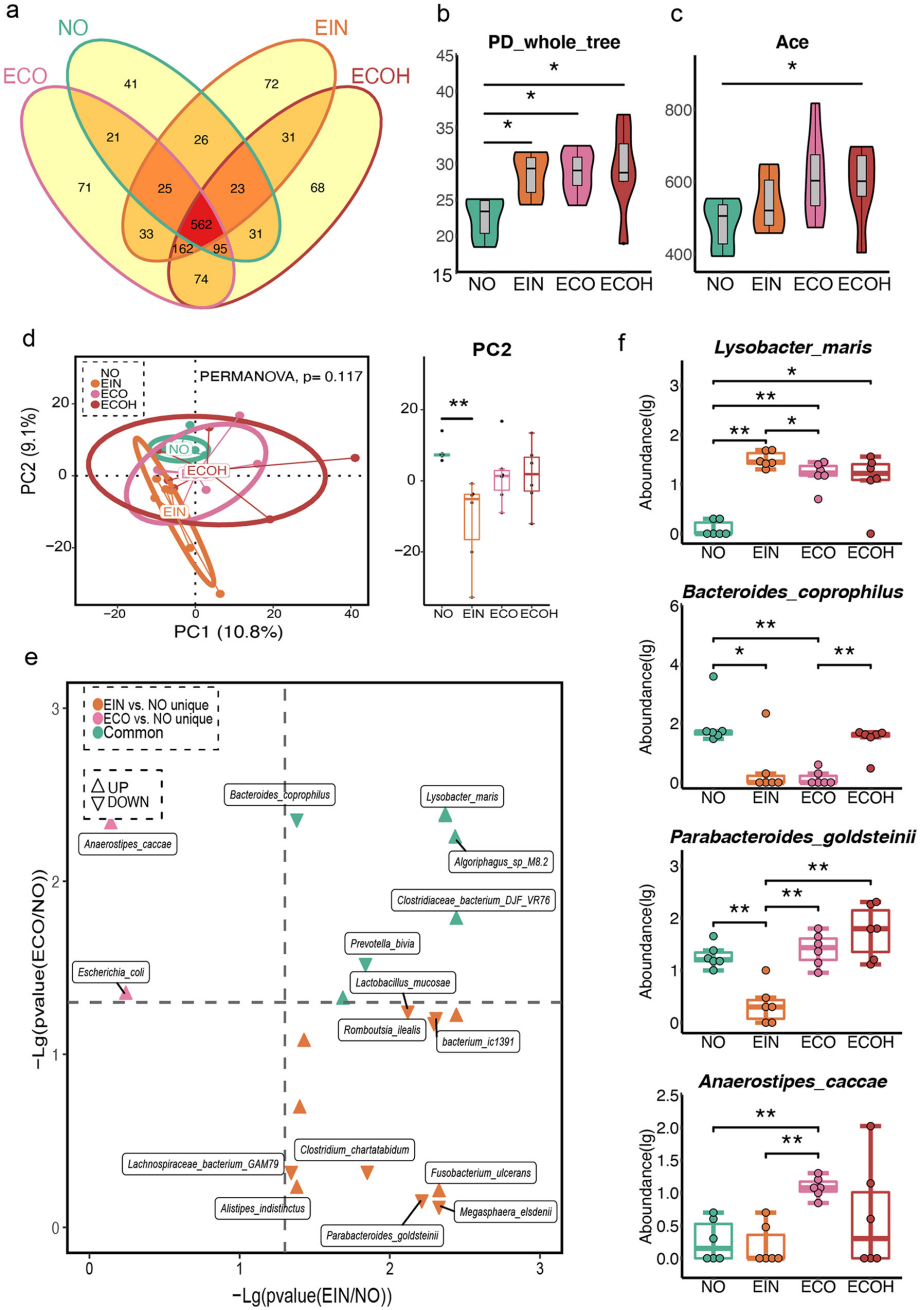
Distribution of intestinal microbiome in overweight subjects. (a) Venn diagram of the observed OTUs in NO, EIN, ECO, and ECOH groups. (b to d) Comparison of alpha- and beta-diversity among four groups. (e) Volcano plots of differential bacterial abundance in EIN, ECO, and the two groups together in comparison to the NO group. (f) Significantly different gut bacteria among four groups (two-tailed Wilcoxon test; *, *P < *0.05; **, *P < *0.01).

To further investigate the characteristics of the microbiota profile in subjects with overweight statuses, we compared bacterial compositions across the NO, EIN, ECO, and ECOH groups. As shown in Fig. 4a, a large proportion of OTUs was shared by these four groups, and the number of unique OTUs in the NO group was lower than that in the other groups. Besides, the number of shared OTUs in the NO, ECO, and ECOH groups was lower than that in the EIN, ECO, and ECOH groups. A remarkably increased alpha-diversity was observed in the EIN, ECO, and ECOH groups compared with that of the NO samples, according to the PD whole tree index (Fig. 4b). Importantly, there was a significant change in the abundance-based coverage (ACE) estimator index between the NO and ECOH groups (Fig. 4c). Even though there were nonsignificant distinct microbiota profiles among the three groups in beta-diversity, a remarkable change between the NO and EIN groups in the PC2 was shown (Fig. 4d). Moreover, a distinct difference was observed between the EIN and ECO groups and the NO group (Fig. 4e). In addition, *Algoriphagus* sp. M8-2 and Lysobacter maris were both increased in the EIN and ECO groups in comparison to NO subjects. Anaerostipes caccae and Bacteroides eggerthii were uniquely increased species in the ECO group in comparison to the NO group. Importantly, Parabacteroides goldsteinii and Megasphaera elsdenii were decreased, but Fusobacterium ulcerans was increased, only in the EIN group. It was noted that even though a decreased level of Bacteroides coprophilus was observed in both the EIN and ECO groups, a higher level of Bacteroides coprophilus was observed in ECOH subjects (Fig. 4f).

Next, we found that the phylum *Proteobacteria* was abnormally increased in the EIN group. However, ECOH subjects demonstrated a higher level of the phylum *Verrucomicrobiota* (Fig. S4a). Furthermore, the genera *Faecalibacterium* and *Dialister* were decreased in the EIN, ECO, and ECOH groups. However, a reverse trend was observed in the *Alistipes* genus (Fig. S4b). In addition, the EIN subjects had the lowest proportion of the genus *Parabacteroides* compared to other groups. It should be noted that a decrease in *Prevotella* was observed in EIN and ECO individuals. *Prevotella* levels were more similar between the NO and ECOH groups. Furthermore, predicted pathways, including those for p53 signaling, the renin-angiotensin system, betalain biosynthesis, and indole alkaloid and isoflavonoid biosynthesis, were enriched in EIN and ECO subjects compared to those in the NO group (Fig. S4c). Furthermore, a correlation analysis was performed to explore the interaction between clinical indices and plasma metabolites. As shown in Fig. S4d, C20, C22, and C22:6 kept a positive association with PAB, albumin (ALB), urea, serum glucose (Glu), and calcium (Ca). And the C20 was positively correlated with total bilirubin (Tbil), direct bilirubin (Dbil), and apolipoprotein a (Apoa), but a reverse association was shown between C22:6 and HDL. It is worth noting that C22 was positively correlated with TG and cholesterol. We further compared the circulating metabolites, and, as shown in Fig. S4e, the plasma C20 and C22 levels were higher in the EIN group than in the NO subjects. In addition, a higher level of C22:6 was observed in ECO individuals. Both plasma alanine and serine levels were decreased in ECOH subjects compared to those in the ECO group. Asparagine was increased in ECO subjects only in comparison with the NO group.

### Combinatorial biomarkers for discriminating EC from normal subjects and cooccurrence analysis among bacteria, metabolites, and clinical indices.

**FIG 5:**
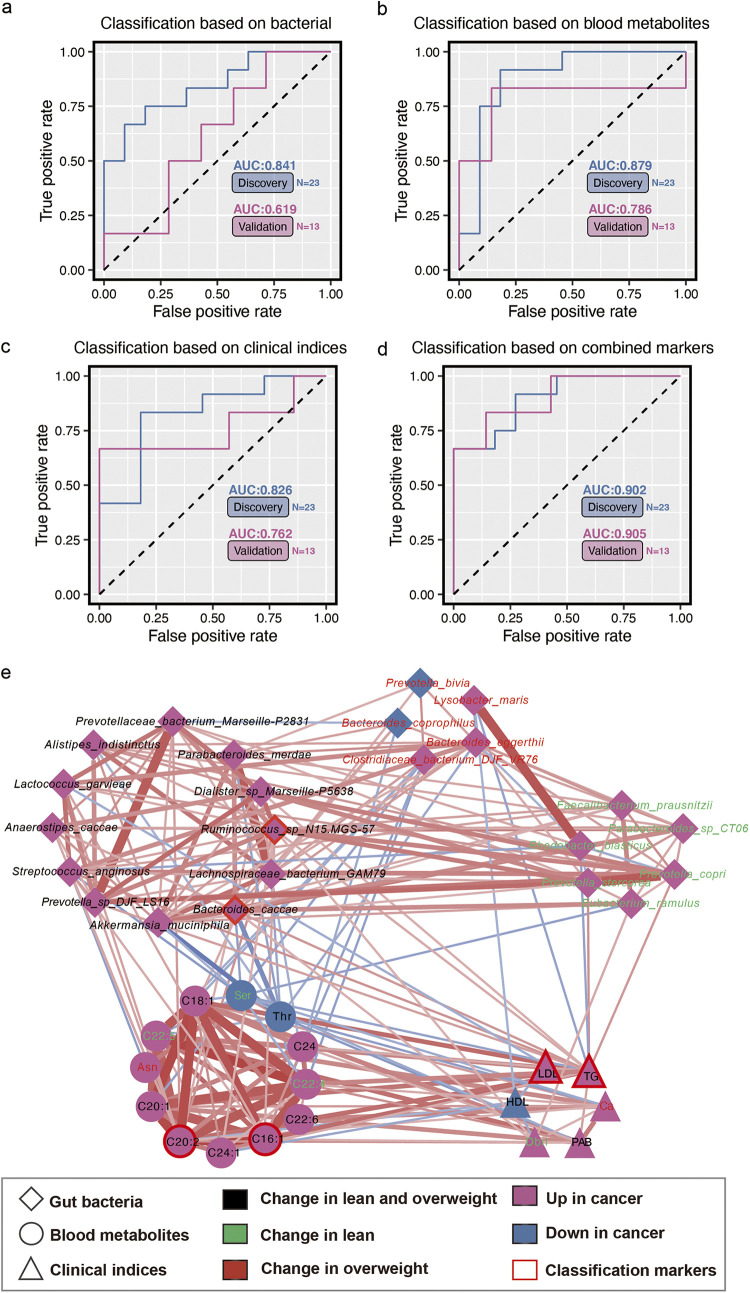
Multiple markers for diagnosis of EC and cooccurrence network. (a to c) Logistic regression models can accurately identify the EC individuals based on fecal bacteria, plasma metabolites, and clinical indices, respectively. (d) This combinatorial marker panel including these 6 markers yielded a more robust diagnostic performance than that of separate markers in both the discovery and validation sets. (e) Cooccurrence network constructed from the different bacterial species, clinical indices, and blood metabolites in EC subjects versus normal individuals. Red and blue dots indicate the increased and decreased relative abundances of variables in EC subjects relative to normal individuals, respectively. Edges between nodes indicate Spearman’s negative (light blue) or positive (light red) correlation; edge thickness indicates the range of correlation values (correlation value of >0.3 or <−0.3).

The potential value of gut microbiome and metabolomic markers was investigated in EC diagnosis using three types of diagnostic models based on differential bacteria, blood metabolites, and clinical indices, respectively. We found that individual marker panels could discriminate normal individuals from EC subjects in both discovery and validation sets (bacterial species, *Ruminococcus* sp. N15.MGS-57 and Bacteroides caccae; plasma metabolites, C16:1 and C20:2; clinical indices, TG and low-density lipoprotein [LDL]) (Fig. 5a to c). Furthermore, a combinatorial marker panel of these biomarkers enabled discrimination of EC from normal subjects with a higher classification power in both the discovery set (AUC = 0.902) and the validation set (AUC = 0.905) (Fig. 5d). As shown in Fig. 5e, a cooccurrence network was utilized to explore the potential interactions among dysbiosis of gut microbiota, serum metabolic patterns, and clinical indices. In this network, these altered metabolites were mainly involved in unsaturated fatty acid metabolism. For example, the increased abundance of species *Ruminococcus* sp. N15.MGS-57 and Bacteroides caccae in all EC patients was strongly positively associated with two potential metabolic markers, C16:1 and C20:2. In addition, Akkermansia muciniphila and Bacteroides caccae were strongly associated with threonine and serine levels, which decreased in EC and lean patients, respectively. More important, the increased level of the species Prevotella copri and Prevotella stercorea only in lean patients was also positively correlated with plasma TG. It is worthwhile to note that the core positions of potential markers imply the importance of these indices in the development of EC.

### Identification and visualization of fatty acids in EC by imaging mass microscopy.

**FIG 6:**
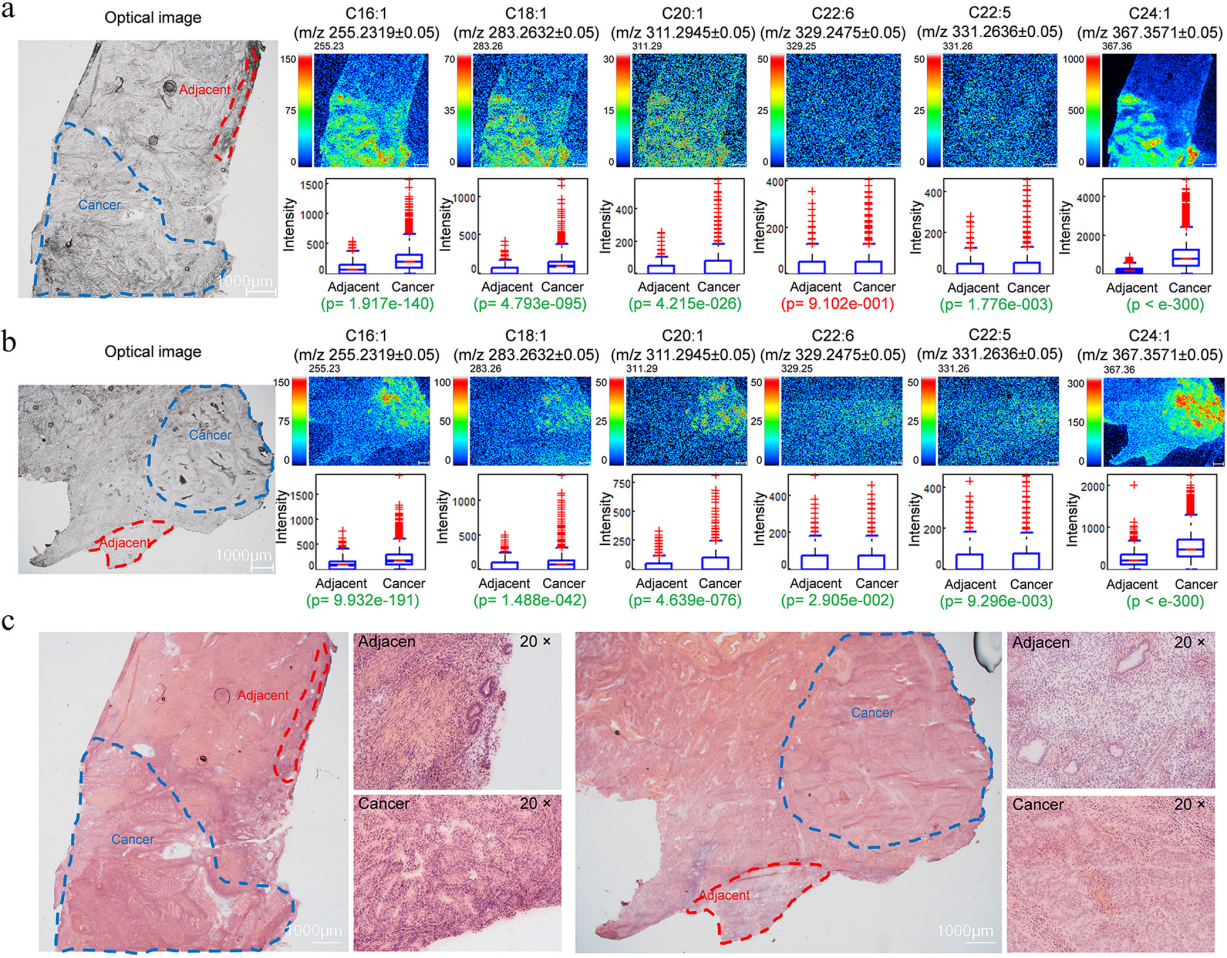
MS imaging-based visual mapping profiles of fatty acids portraying the distribution of the indicated fatty acid ion species in tumor tissue and adjacent tissue in two EC patients. (a and b) Distribution profiles of fatty acids in EC tumor tissues were studied through comparisons of relative intensities of fatty acids by *P* values between adjacent and cancer tissues. Signal intensities of targeted fatty acids in adjacent and cancer tumor tissues of EC patients are depicted, was normalized by measuring pixel per 100 μm. “p” denotes the statistical *P* value of the comparison. Significant differences are highlighted in green font, while insignificant differences are highlighted in red font. (c) H&E staining of EC tumor tissues from these two EC patients.

The distributions of fatty acids in EC tissues were visualized by the novel iMScope with a mass spectrometry (MS) imaging technology. Direct detection of fatty acids obtained from EC tissues was made possible without the utilization of antibodies or extensive purification steps. Tissue frozen sections from two individuals showed the representative optical microscopic image and MS visual mappings of fatty acids on EC tissue and adjacent tissue (Fig. 6a and b). According to the results of metabolomics, the differential targeted fatty acids include C16:1, C18:1, C20:1, C22:6, C22:5, and C24:1. The localizations of these fatty acids were distributed mostly over the area of cancer tissue. To evaluate the relative abundance and distribution of detectable fatty acids in the sampled tumor tissues, we further analyzed the mass spectral data by analyses of detectable fatty acids in the region of adjacent tissue and cancer tissue. As can be seen from the results, significantly higher levels of fatty acid expression in cancer are consistent with the metabolomics, especially in C16:1 and C18:1. Sectioned tissues detected by iMScope were subjected to hematoxylin and eosin (H&E) staining to identify the localization of adjacent tissue and cancer tissue in EC (Fig. 6c). H&E staining more discriminately demonstrates the adjacent tissue and cancer boundaries, which are highly consistent with the location and distribution of high fatty acid aggregation in EC determined by iMScope. Combining high-resolution MS with *in situ* spatial analysis of the area, abnormalities in fatty acid synthesis and uptake were clearly observed in EC.

### Altered metabolites contribute to EC cell viability and mTOR pathway activity.

**FIG 7:**
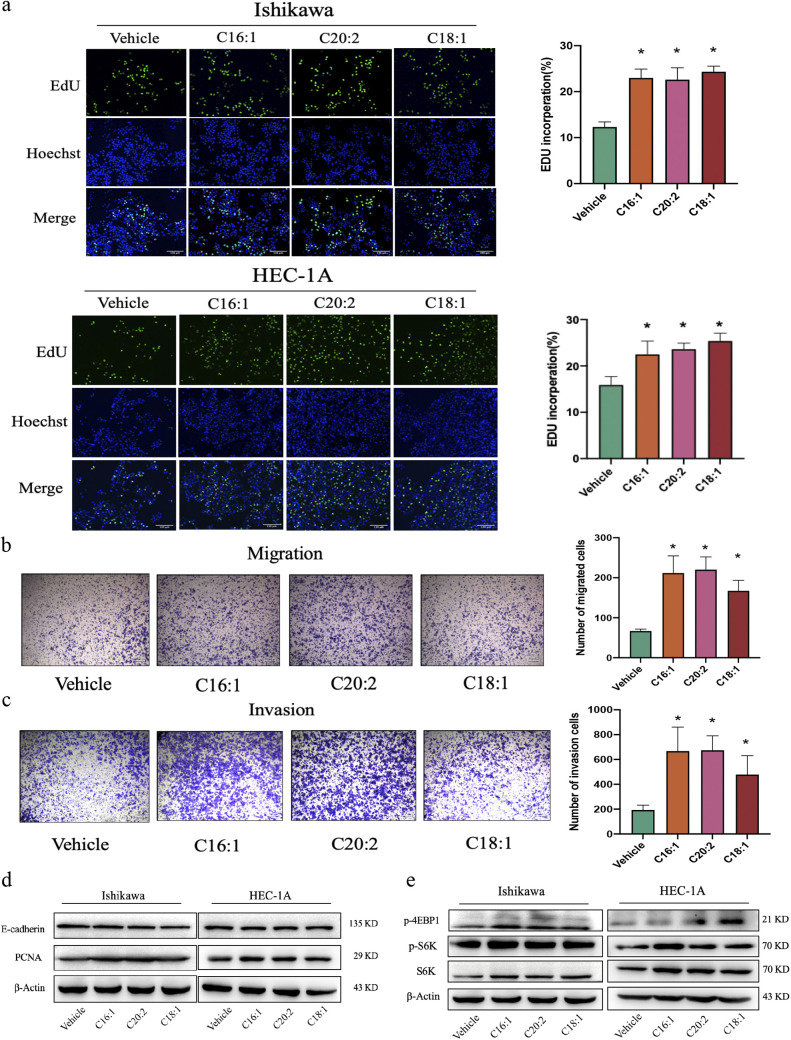
C16:1, C20:2, and C18:1 supplementation promoted EC proliferation, migration, and invasion *in vitro*. (a) Percentage of EdU-positive Ishikawa and HEC-1A cells after C16:1, C20:2, and C18:1 treatment at 5 μΜ for 24 h by EdU staining (magnification, ×100). Data are shown as the mean ± SD (*, *P* < 0.05). (b and c) Migration (b) and invasion (c) assays were performed by transferring Ishikawa cells to serum-free medium in the absence or presence of C16:1, C20:2, and C18:1 (5 μΜ) in inserts with 8-μm-pore-size membranes coated with Matrigel or not. Migration and invasion times were 12 h and 24 h, respectively. Cell numbers are given as the average number ± SD per field and were counted at ×100 magnification (*n* = 6). *, *P < *0.05 compared with controls. (d) Immunoblotting analysis of PCNA and E-cadherin in Ishikawa and HEC-1A cells treated with or without C16:1, C20:2, and C18:1 (5 μM) for 24 h. β-Actin served as an internal control. (e) Immunoblotting analysis of S6K, P-S6K, and P-4EBP1 in Ishikawa and HEC-1A cells treated with or without C16:1, C20:2, and C18:1 (5 μM) for 24 h. β-Actin served as an internal control. Results are representative of at least three independent experiments.

To confirm the causal relationship between fatty acid metabolite disorder and EC, cells were treated with olive oil for 24 h. The results showed that olive oil significantly stimulated Ishikawa cell proliferation in a dose-dependent manner (Fig. S5a). To further investigate the results of metabolome and iMScope analyses in EC, we tested the modulation of cell function by C16:1, C20:2, and C18:1, the most relevant fatty acids *in vitro*. C16:1, C20:2, and C18:1 stimulated cell proliferation, with the highest induction by C16:1 in both Ishikawa and HEC-1A cells (Fig. S5b). Additionally, C16:1-, C20:2-, and C18:1-treated EC cells formed a higher number of replicated DNA than the vehicle control (Fig. 7a), which revealed the improved survival and proliferative capacity of cells incubated with C16:1, C20:2, and C18:1. Cell migration and invasion ability were also significantly increased by C16:1, C20:2, and C18:1 (Fig. 7b and c). In addition, the epithelial-mesenchymal transition (EMT) and cell invasion marker PCNA were examined with or without fatty acids in Ishikawa and HEC-1A cells by immunoblotting (Fig. 7d). These data demonstrated that C16:1, C20:2, and C18:1 have tumor-promoting effects *in vitro*. Oncogenic signaling and metabolic alterations are interrelated in cancer cells. mTOR is frequently activated in cancer and controls cell growth and metabolism. mTOR signaling regulates amino acid, glucose, nucleotide, fatty acid, and lipid metabolism. We analyzed the levels of downstream target proteins of mTOR in EC cells by immunoblotting. Compared with the findings for the control group, the analysis showed increased phosphorylation of S6K and 4EBP1 at the protein level after treatment (Fig. 7e).

## DISCUSSION

The connection between gut microbiota and endometrial cancer in humans, to our knowledge, has been identified for the first time. *Ruminococcus* sp. N15.MGS-5 and metabolic products C16:1 and C20:2 demonstrate similar rising trends from normal subjects to EC patients. In addition, *Ruminococcus* sp. N15.MGS-5 contributed significantly to the EC group separation and was significantly increased in ECC individuals compared to the NC group. Meanwhile, the increased abundance of *Ruminococcus* sp. N15.MGS-5 in all EC patients was strongly positively associated with C16:1 and C20:2. The localization of C16:1 was identified and spatially visualized by imaging mass microscopy, and C16:1 showed a high distribution within the tumor area in comparison to that in adjacent tissues. Further study confirmed that C16:1 may stimulate Ishikawa and HEC-1A cell proliferation through the mTOR pathway.

Here, the connection between the gut microbiota, obesity, and fatty acid metabolism has been identified, and it strongly indicates a key role of the microbiome in the etiology of EC. The current epidemiological evidence clarifies the relationship between obesity and EC incidence and mortality risk. Obesity, measured and defined by BMI (>30 kg/m^2^ and <35 kg/m^2^), was associated with a 2.6-fold increase in EC risk, while severe obesity (BMI, >35 kg/m^2^) was associated with a 4.7-fold increase in risk compared to women of normal weight (BMI, <25 kg/m^2^) (14). Obesity is hypothesized to increase EC risk, including increased endogenous sex steroid hormones. Research has confirmed that estrogen plays a crucial role in the development of EC. Hormonal imbalance, especially adipose-derived unopposed estrogen in obese postmenopausal women, is the most established. The increased amount of adipose tissue in obesity results in an increased level of estrone conversion. Estrogen, by binding to endometrial cell DNA, activates the proliferative PI3K/AKT/mTOR signaling pathway and also positively modulates the expression of genes linked to endometrial proliferation, leading to uncontrolled cellular proliferation and the accumulation of replication errors that predispose to malignancy (15). Our results showed that the β-activity of gut microbiota based on the unweighted UniFrac of ECC subjects was significant lower than that of NC subjects, indicating a more similar community structure among EC patients. Furthermore, beta-diversity trended higher with increasing comorbidities, which seemed to further explain the role of gut microbiota in EC progression. At the phylum level, the abundances of *Bacteroidota*, *Verrucomicrobiota*, and *Firmicutes* were higher, while the abundance of *Proteobacteria* was lower in patients with EC. The members of *Bacteroidota* have been reported to be associated with immunity and metabolic processes. *Bacteroidota* interact with the host by glycoprotein secretion, short fatty acid imbalance, toxin production, and molecular mimicry, which are involved in many diseases, such as autoimmune diseases, metabolic syndrome diseases (obesity, diabetes mellitus, atherosclerosis, hypertension), and neurodegenerative disorders (16). The metabolic syndrome can not only predict endometrial cancer risk occurances, but also be associated with worse overall and disease-free survival in endometrial cancer survivors. By analyzing the association between clinical indicators and gut microbiota, we found that in comparison with the normal group, triglycerides increased and high-density lipoprotein decreased in the EC group. Significantly, the abundance of *Ruminococcus* sp. N15.MGS-5, which increased in EC subjects, was positively associated with serum TG and negatively with HDL. According to the International Diabetes Federation and the WHO, increased TG and reduced HDL are components of metabolic syndrome. High levels of serum TG can further accumulate adipose tissue, convert androgens into estrogen under the action of aromatase, and further increase the level of estrogen in the body. A previous study used genetic markers to predict low-density lipoprotein (LDL) and HDL cholesterol levels and to analyze EC risk. When lower LDL or higher HDL levels were predicted, EC risk was increased (17). Thus, our results indicate that *Ruminococcus* sp. N15.MGS-5 may participate in EC development through HDL and TG metabolism. These data clearly show the connection between gut microbiota and EC, and the microbiome could be a potential regulator and therapeutic target of EC.

The gastrointestinal tract microbiota in adults is dominated by two divisions of bacteria, the phyla *Firmicutes* and *Bacteroidota* (18). The *Firmicutes* are composed mainly of the class *Clostridia*, which is divided into three major *Clostridium* clusters, IV, IX, and XIV. The genus *Ruminococcus* falls into *Clostridium* clusters IV and XIVa and is defined as strictly anaerobic, Gram-positive, and nonmotile cocci (19). *Ruminococcus* plays an important role in the digestion of resistant starch, but it is also associated with intestinal diseases (irritable bowel syndrome [IBS], irritable bowel disease [IBD], and Crohn's disease), immune diseases (allergies, eczema, and asthma), nervous system diseases (autism and depression), and metabolic diseases (obesity and diabetes) (20–22). Some studies have also found a relationship between *Ruminococcus* and cancer. *Ruminococcus* plays an essential role in prostate cancer progression, possibly through the activation of the “*Ruminococcus*-LPCAT1-DNA repair” pathway (23). The relative abundance of *Ruminococcus* is also higher in lung cancer patients (24). FishTaco analysis identified *Ruminococcus* and *Coprococcus* as the taxa potentially contributing to enriched KEGG pathways for the biosynthesis of amino acids and to the metabolisms of pyruvate, glycerophospholipid, and nicotinate and nicotinamide (25). Notably, the involvement of *Ruminococcus* in lipid metabolism has been reported in several studies (26). Bacteroides caccae is a ubiquitous, anaerobic bacterium and is regarded as an opportunistic pathogen. It can invade the mucosa of the intestine and cause various infections (27). Many clinical studies have confirmed the association between metabolic syndrome and endometrial cancer (28). Obesity and the metabolic syndrome are associated with microbiota alterations. The gut microbiota has also been related to the development of obesity, and it is known that overweight and obese women have a higher risk of EC than women of normal weight, especially during the postmenopausal period (29). The presence of *Ruminococcus* also differed in the EC versus normal uteri in obese mice (*P < *0.05). These data suggest that the microbiome may play a role in obesity-driven EC (30). Based on the International Agency for Research on Cancer (IARC) Working Group, there is convincing evidence that excess body weight is associated with an increased risk for cancer in at least 13 anatomic sites (31). In addition, the higher intake of palmitic acid could increase the abundance of intestinal *Ruminococcus* sp. (32). Thus, our data indicate that lipids may play a key role in EC development through the microbiome.

With reference to the results for the above-described intestinal bacteria and clinical tests and in order to further study the relationship between gut microbiota and lipid metabolism, we quantified plasma metabolites using targeted metabolomics. By integrating data on the host gut microbiome, and fasting serum metabolome, we were able to demonstrate clear metabolome signatures of EC phenotypes distinguished from those of the normal control. The EC-associated metabolome associates with functional components of the EC-linked gut microbiome, notably the increase for fatty acid biosynthesis. Numerous studies have since confirmed the importance of fatty acids for cancer cell growth and survival (33). Several blood-based EC diagnostic metabolomic biomarkers have been reported in the literature, mostly byproducts of lipids and amino acids. They include acylcholines, monoglycerols, acylcarnitines, phenylalanine, phosphocholine, modified phosphatidylcholine derivatives, lactic acid, progesterone, indole acetic acid, homocysteine, stearic acid, valine, tetradecadienoylcarnitine, 3-hydroxybutyric acid, proline/tyrosine, and lyso-platelet-activating factor-16, among others (34). Eicosanoids (C20) comprise a diverse group of bioactive lipids which orchestrate inflammation, immunity, and tissue homeostasis and whose dysregulation has been implicated in carcinogenesis. Eicosanoid metabolism through the COX-2/PGE2 axis is associated with malignant transformation. The eicosanoid metabolic enzyme gene *HPGD* combined with *ALOX5* expression has been associated with the worst overall and progression-free survival in type II EC (35). Research has found that high saturated fatty acid intake could increase cancer risk, especially breast cancer (36). Experimental studies addressing the effects of olive oil on cancer progression seem to show protective effects (37). However, inconsistent data showing a tumor-enhancing role of oleic acid (C18:1) in many cancer types have also been reported (38, 39). Studies have shown that dietary palmitic acid (C16:0) promotes metastasis in oral carcinomas and melanomas in mice (40). Tumors from mice that were fed a short-term palm oil-rich diet, or tumor cells that were briefly exposed to palmitic acid *in vitro*, remained highly metastatic even after being serially transplanted. Thus, fatty acid intake and metabolic alterations are important features of cancer development (41). In the nonalcoholic fatty liver disease-associated hepatocellular carcinoma model, increased palmitic acid enhanced the protein expression of endoplasmic reticulum-activating oncogenic JNK/c-jun/AP-1 and NF-κB cascades (42). In addition, palmitic acid or high-fat diet specifically enhances the metastatic potential of CD36^+^ metastasis, initiating cells in a CD36-dependent manner (43). Enhanced palmitic acid intake could increase the abundance of intestinal *Ruminococcus* sp. (32), and the isolated *Ruminococcus* sp. from human feces contains a high proportion of C16:1 and C18:1 (19). In our study, we found that C16:1, C18:1, C20:1, C20:2, C22:6, C24, and C24:1 in plasma were enriched in EC subjects and related to *Ruminococcus* sp, N15.MGS.57. Metabolic reprogramming has been recognized as a new hallmark of tumorigenesis. Spatial visualization of fatty acids showed that C16:1 and C18:1 accumulate obviously in tumor tissue and that C16:1 may promote EC cell invasion and metastasis through mTOR signaling. Nutrient sensing plays a major role in the activation of mTOR1, and mTOR drives cancer metabolic reprogramming. More mechanisms to explore this mutual determinism can be better used in cancer treatment. Although we found the correlation between gut microbiota and fatty acids in EC patients, the sample size was small. A large-scale screening should be the subject of future studies. The gut microbiota has changed significantly, but further mechanism research is still needed. For instance, experiments are needed to validate screening for unique microbes that can serve as markers in vitro and in vivo. We remain interested in the relationship between the microbiota of the gastrointestinal and female reproductive tracts in EC. Metagenome analysis with more sophisticated analytical capabilities will provide more detailed information to closely explore intestinal microbiota disorders.

### Conclusion.

For the first time, the differential profiles of gut microbiota between EC patients and normal participants, as well as the association between gut microbiota and EC, have been identified. EC patients showed clear dysbiosis in comparison to normal individuals. The trend of intestinal microbiota from normal individuals to precancer to EC can potentially reveal the process of cancer. Gut microbiota dysbiosis may contribute to metabolomic dysbiosis and fatty acid elevation, leading to endometrial cancer. Thus, fatty acid-associated endometrium tumorigenesis is correlated with gut microbiota. Our study found that *Ruminococcus* sp. N15.MGS.57 and C16:1 showed prominent positive significance in the results of each experiment. The aberrant fecal microbiome, more specifically, *Ruminococcus* sp. N15.MGS.57 and spatially distributed C16:1 in EC tissues, can be used as a biomarker of clinical features and outcomes and provide a new therapeutic target for clinical treatment. This study shows that changes in fecal microbiota and blood biochemical indexes may be used to predict and characterize the development of metabolic abnormalities in endometrial cancer.

## MATERIALS AND METHODS

### Participant information.

All participants were recruited from the Cancer Hospital of China Medical University, Liaoning Cancer Hospital & Institute, from September 2019 to December 2020. The study and experimental procedures were approved by the Ethics Committee of Liaoning Cancer Hospital & Institute (no. 20180903). All participants signed a written informed consent upon enrollment. Patients were eligible if they had an initial diagnosis of EC or benign disease and had undergone curettage or hysterectomy. Exclusion criteria included previous pelvic radiotherapy, hormonal therapy, or chemotherapy, patients with previous or combined other malignant tumors, and incomplete clinical data and follow-up. Information about height, body weight, menstruation, histological type, health status, and laboratory results was recorded. Body mass index was derived as the weight (in kilograms) divided by the square of the height (meters).

### Sample collection.

Blood samples were collected after fasting in the morning using a K_2_EDTA anticoagulant tube (BD Vacutainer). The samples were centrifuged at 3,000 rpm for 15 min at 4°C. After centrifugation, the samples were immediately frozen to −80°C until the time of analysis. Fecal samples were collected by each participant with fecal collection containers and then frozen at −20°C. The samples were transferred to the laboratory and stored at −80°C until DNA extraction. Tissue samples of EC and normal endometrium were collected from patients with EC and patients with benign gynecological diseases who had undergone surgical resection.

### Clinical index measurements.

Inpatient laboratory results data of enrolled individuals were collected. Triglyceride (TG), serum glucose (Glu), alanine aminotransferase (ALT), aspartate aminotransferase (AST), creatinine (Cre), urea, cholesterol, retinol-binding protein (RBP), prealbumin (PAB), cholinesterase (ChE), albumin (ALB), cystatin c (Cys c), cholylglycine (CG), and high-density lipoprotein (HDL) were detected by using an autoanalyzer.

### DNA extraction and 16S rRNA sequencing.

The total genome DNA of stool samples (100 mg per sample) was extracted using the hexadecyltrimethylammonium bromide (CTAB) method. DNA purity was monitored on 1% agarose gels. In addition, the concentration of DNA was detected using a Qubit 2.0 fluorometer (Thermo Scientific, USA). DNA was diluted to l μg/μL using sterile water. The V4 hypervariable regions of the bacterial 16S rRNA gene were amplified using specific primers, 515F (GTGCCAGCMGCCGCGGTAA) and 806R (GGACTACHVGGGTWTCTAAT). All PCRs were carried out with 15 μL of Phusion high-fidelity PCR master mix (New England Biolabs), 0.2 μM concentrations of forward and reverse primers, and about 10 ng template DNA. Thermal cycling consisted of initial denaturation at 98°C for 1 min, followed by 30 cycles of denaturation at 98°C for 10 s, annealing at 50°C for 30 s, and elongation at 72°C for 30 s. A final step of 72°C for 5 min followed. PCR products were purified with a Qiagen gel extraction kit (Qiagen, Germany). Sequencing libraries were generated using a TruSeq DNA PCR-free sample preparation kit (Illumina, USA) in accordance with the manufacturer's recommendations, and index codes were added. The library was sequenced on an Illumina HiSeq platform (Novogene, China), and 250-bp paired-end reads were generated by using FLASH (version 1.2.7).

### Intestinal microbiota diversity analysis.

Paired-end reads were assigned to each sample according to the unique barcodes and were truncated by cutting off the barcode and primer sequence. After being filtered by QIIME quality filters, sequences with ≥97% similarity were clustered into the same operational taxonomic units (OTUs) by Uparse software (version 7.1). A representative sequence for each OTU was annotated with taxonomic information by the Ribosomal Database Project (RDP) classifier algorithm according to the Silva database. QIIME (version 1.9.0) was used to analyze alpha (within-sample)- and beta (among-sample)-diversity.

### Analysis of flora structure and predictive function.

Principal-component analysis (PCA) was used to reveal the differences in intestinal microbiome profiles, which was performed by using the R package factoextra. Analysis of similarities (PERMANOVA) among groups was analyzed by using the R package vegan. The Kruskal-Wallis test was used for the two principal components obtained from PCA. In addition, a representative sequence of OTUs in individuals was used to predict the function of the intestinal microbiome by PICRUST2 and visualized by the R package ggplot2. Finally, we used the R package cyclize to reveal the identifiable distribution of the microbiome in individuals, which was shown in a circle plot.

### Targeted metabolomics quantification of plasma metabolites.

Plasma concentrations of 20 amino acid species and 18 fatty acids of subjects were quantified using previously described methods (44). Briefly, amino acids and fatty acids were quantified by high-performance liquid chromatography (HPLC) coupled to tandem mass spectrometry (MS/MS) based on deuterated purified standards. Plasma amino acid and fatty acid concentrations were expressed in micromoles per liter and nanomoles per liter, respectively.

### Visualization of fatty acid distribution by iMScope.

Fatty acid distribution in EC tissue was quantified and visualized by using an iMScope. The method was developed based on our previously described method (45). In brief, the frozen EC tissues were cut at 10 μm, and specimens were mounted on electrically conductive glass slides. Subsequently, a “two-step matrix application,” which combined sublimation and airbrushing, was used to coat the a-cyano-4-hydroxycinnamic acid (CHCA, no. C2020) matrix for tissue sections. The parameters of iMScope were set as follows: frequency, 1,000 Hz; laser intensity, 55.0; laser diameter, 3 μm; ion polarity, positive; mass range, 200 to 400; sample voltage, 3.5 kV; and detector voltage,1.85 kV. The Imaging Mass Solution version 1.30 software (Shimadzu, Tokyo, Japan) was used to control the instrument, and data acquisition, visualization, and quantification were also performed by the same software. The *m/z* values were externally calibrated using a 2, 5-dihydroxybenzoic acid (DHB, no. 149357) matrix. Three serial sections (each measuring 10 μm) of sampled tumor tissues were used to evaluate the reproducibility of the iMScope technique.

### Cooccurrence network analysis.

The species cooccurrence network was constructed by using R package Hmisc, and correlation was visualized by using Cytoscape 3.8.2. In addition, the unique and shared edges were counted by the R package Vennerable. Meanwhile, Spearman’s correlations among the gut microbiota, clinical indexes, gut microbiota, and KEGG pathways were determined using the R package psych.

### Identification of biomarker species for different healthy statuses.

Partial least-squares discriminant analysis (PLS-DA) was used to reveal the taxonomic changes in groups by using R package ropls and the significant microbiome with a VIP score of ≥1, and the corresponding phylum was visualized by using R package ggplot2.

### Marker panel for EC.

Random forest analysis was used to quantify the diagnostic performance of this microbial marker panel with the AUC in the validation set. Based on the three types of marker panels, logistic regression models were built to identify the EC samples with the AUC in both the discovery and validation sets as previously described.

### Cell culture and treatment.

EC cell lines Ishikawa and HEC-1A were obtained from the Cancer Hospital of China Medical University. Ishikawa cells were cultured in Dulbecco modified Eagle medium (DMEM) containing 10% fetal bovine serum (FBS) and 1% penicillin-streptomycin. HEC-1A cells were cultured in McCoy’s 5A medium containing 10% FBS and 1% penicillin-streptomycin. Both cultures were maintained in a humidified 5% CO_2_ atmosphere at 37°C. For cell treatment, Ishikawa and HEC-1A cells were incubated in the presence of fatty acids. The same volume of dimethyl sulfoxide was used as the vehicle control.

### Cell proliferation assay.

Ishikawa and HEC-1A cells were seeded in 96-well plates at a density of 5,000 cells/well and incubated in normal growth medium. After 1 day, the cells were subjected to C16:1, C18:1, and C20:2 loading (from 0 to 5 μM) for 24 h. All experiments were carried out in serum-free medium. The optical density (OD) values were measured at 450 nm after incubation with CCK-8 reagent for 1 h at 37°C.

### Immunoblotting.

Ishikawa and HEC-1A cells were lysed in radioimmunoprecipitation (RIPA) buffer containing complete protease inhibitor cocktail. The lysates were incubated for 20 min on ice and centrifuged at 12,000 × *g* for 15 min at 4°C. Protein concentration was measured using the bicinchoninic acid (BCA) protein assay. The lysates were boiled in sodium dodecyl sulfate (SDS) Laemmli sample buffer for 10 min, resolved using SDS-polyacrylamide gel electrophoresis, transferred to polyvinylidene difluoride (PVDF) membranes (Millipore), and probed with primary antibodies against PCNA, E-cadherin, p-4EBP1, p-p70S6K, p70S6K, and β-actin (Cell Signaling Technology) secondary antibodies for 1 h. Bands were detected by using an enhanced chemiluminescence kit (Advansta) and visualized by detection using the Tanon-5200 multiautomatic chemiluminescence fluorescence image analysis system.

### EdU labeling assay.

EdU labeling assays were performed using BeyoClick EdU-488 assay kits (Beyotime). Cells were seeded in 24-well plates at 2 × 10^4^ cells per well. After 24 h of the indicated treatment, each well was incubated with EdU medium for 2 h. The cells were fixed in phosphate-buffered saline (PBS) containing 4% paraformaldehyde for 15 min and then washed with PBS containing 0.3% Triton X-100 for 10 min. After washing again with PBS, 1× Hoechst 33342 was added, and the mixture was incubated for 10 min at room temperature. After washing with PBS, the cells were observed by fluorescence microscopy.

### Statistical analysis.

A two-tailed Wilcoxon test was performed to analyze differences between the two groups. For statistics in multiple groups, we utilized the Kruskal-Wallis analysis of variance (ANOVA) test to evaluate the differences among groups. *P* values of <0.05 were considered statistically significant. Error bars indicate mean ± standard deviation (SD). The region of interest (ROI) analysis was performed using Imaging Mass Solution software (version 1.30). The *P* value of comparisons for ROI analyses was assessed via the average peak intensities or signals acquired from MS spectra of areas indicated by ROI. Low *P* values (<0.05) denote significant differences between average peak intensities or signals of targets within the stipulated ROIs.

### Data availability.

The data that support the findings of this study are available from the corresponding author, H.-X.L., upon reasonable request. The 16s rRNA raw data have been submitted to the SRA database under BioProject accession number PRJNA833670.
