# Supplementary material for: Altered Gut Microbial Profile Accompanied by Abnormal Fatty Acid Metabolism Activity Exacerbates Endometrial Cancer Progression
Source: Microbiol Spectr. 2022 Oct 13;10(6):e02612-22. doi: 10.1128/spectrum.02612-22 (PMC9769730; doi:10.1128/spectrum.02612-22)
Supplement: Supplemental file 1 — Fig. S1 to S5. Download spectrum.02612-22-s0001.pdf, PDF file, 2.0 MB [file spectrum.02612-22-s0001.pdf]

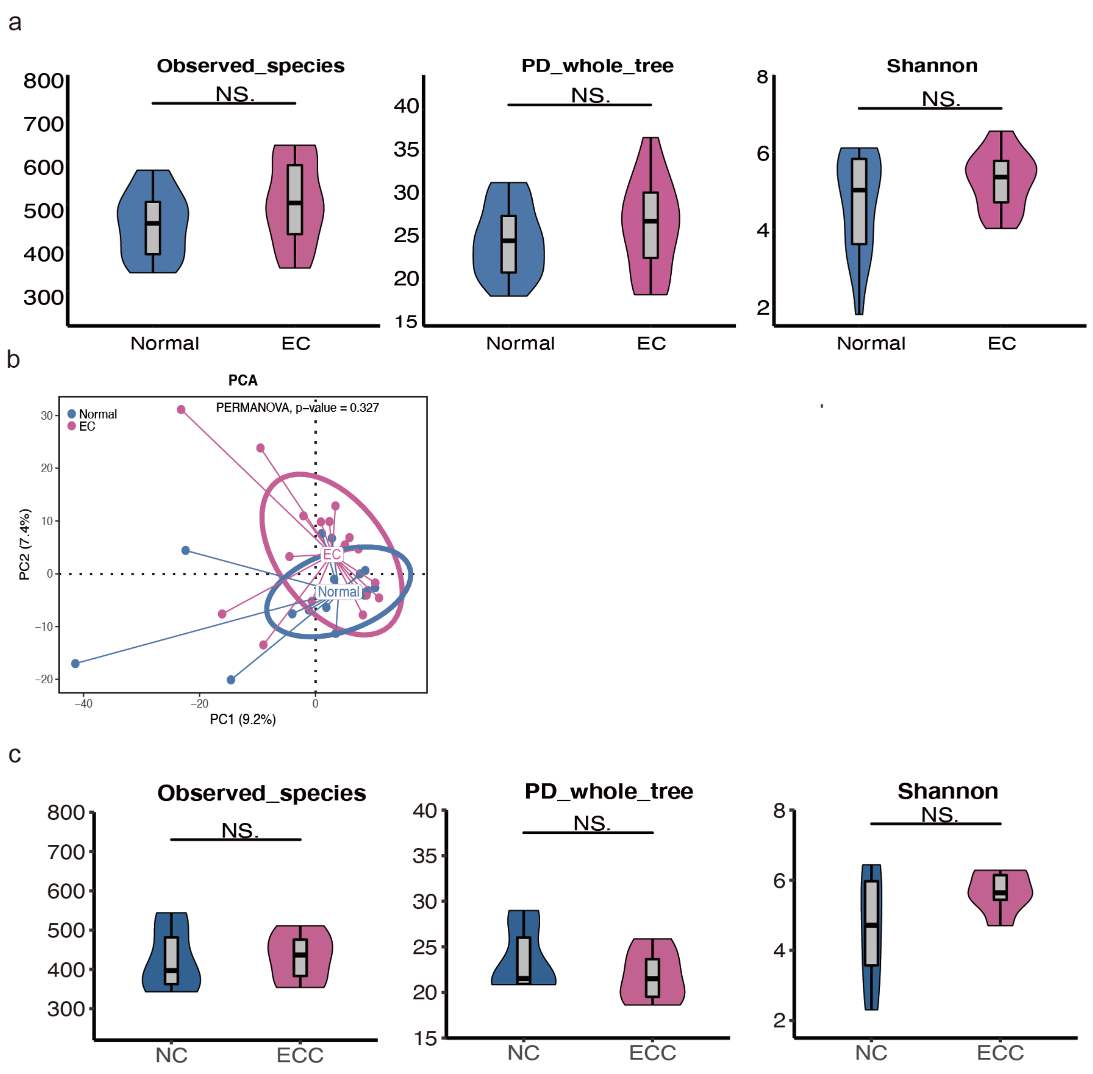

Supplementary figure S1. The change of intestinal microbiota in EC and Normal individuals. (a) Alpha diversity in subjects with different status. (b) Beta diversity in EC and Normal. (c) Alpha diversity in NC and ECC groups.

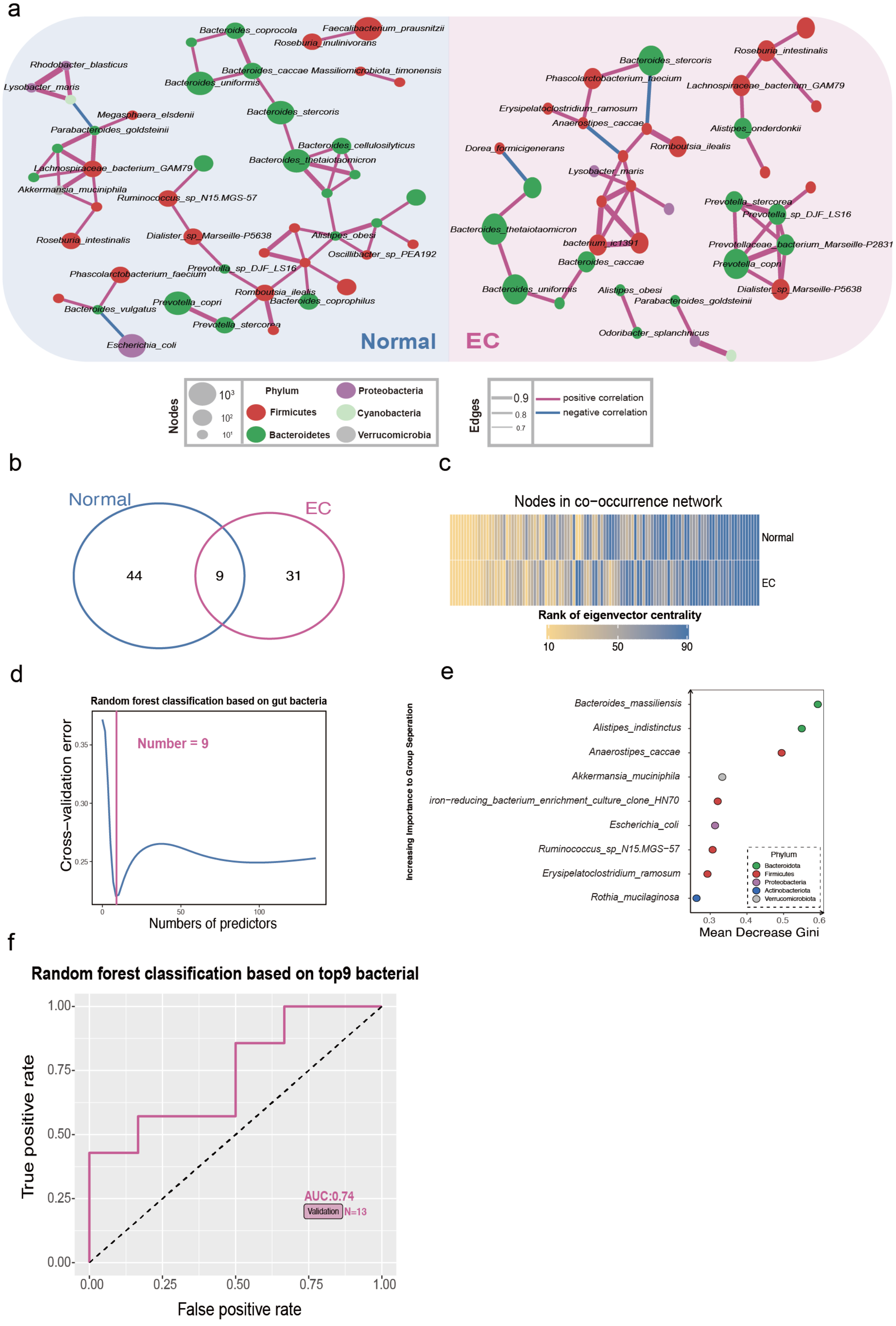
a
Normal
EC
Nodes
Phylum
Proteobacteria
Firmicutes
Cyanobacteria
Bacteroidetes
Verrucomicrobia
Edges
positive correlation
negative correlation
b
Normal
EC
44
9
31
c
Nodes in co−occurrence network
Normal
EC
Rank of eigenvector centrality
10
50
90
d
Random forest classification based on gut bacteria
Number = 9
Cross−validation error
Numbers of predictors
e
Increasing Importance to Group Seperation
Bacteroides_massiliensis
Alistipes_indistinctus
Anaerostipes_caccae
Akkermansia_muciniphila
iron−reducing_bacterium_enrichment_culture_clone_HN70
Escherichia_coli
Ruminococcus_sp_N15.MGS−57
Erysipelatoclostridium_ramosum
Rothia_mucilaginosa
Phylum
Bacteroidota
Firmicutes
Proteobacteria
Actinobacteriota
Verrucomicrobiota
Mean Decrease Gini
f
Random forest classification based on top9 bacterial
True positive rate
False positive rate
AUC:0.74
Validation
N=13

Supplementary figure S2. The strikingly different intestinal microbiota structure between normal and EC subjects. (a) Co-occurrence networks of intestinal microbiota of Normal (left panel) and EC (right panel). The co-occurrence network was inferred for each sample type by a pairwise correlation of abundance for all species. Each node in the network indicates a bacterial species. Node size represents the average abundance of one species in each sample type. Only the bacterial connections (edges) larger than cut-offs (correlation values >0.7 and FDR<0.05 in the network) are retained. Edge width represents the correlation value supporting this connection. (b and c) Discrepancies of the bacterial co-occurrence networks between Normal and EC. The number of unique and shared edges, and eigenvector centrality of nodes in normal and EC co-occurrence networks were counted, respectively. (d) Classification performance of a random forest model using species abundance assessed by R random Forest package. The cross-validated prediction performance of models with sequentially reduced number of predictors was explored and ordered by importance. (e) The 9 most discriminant species in the models classifying Normal and EC. (f) ROC curve of random Forest models classifying Normal, and EC based on the 9 most discriminant species in the validation set.

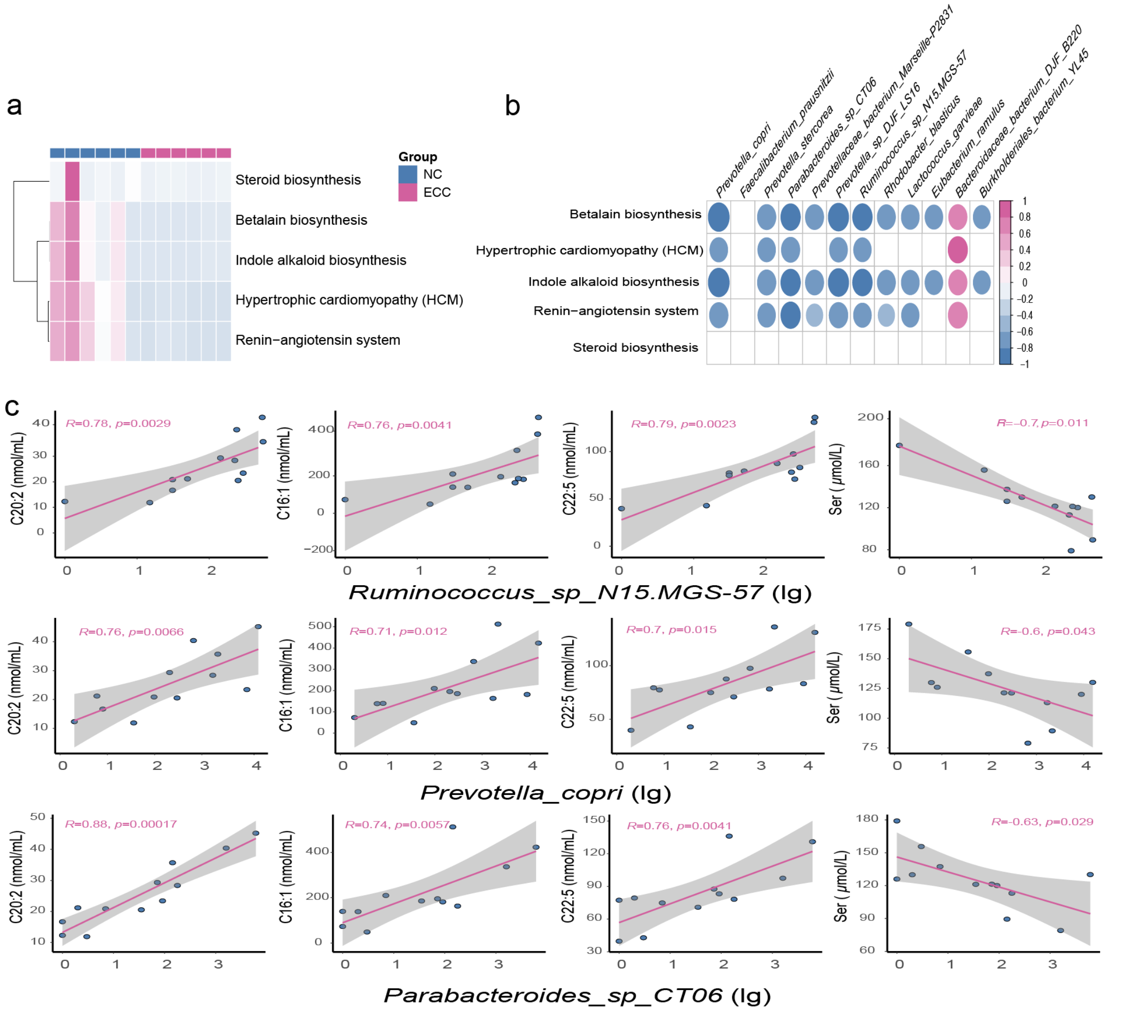

Supplementary figure S3. The function of gut microbiota and metabolic characteristic in ECC patients. (a) Predicted abundance of KEGG pathways according to intestinal microbiota at level 3 by PICRUST2 (only pathways with significant difference between two groups are shown. (b) Correlations between significantly changed species and KEGG pathways. Only correlations with $p < 0.05$ were indicated with colored circles. (c) The correlation between altered metabolites and gut bacterial.

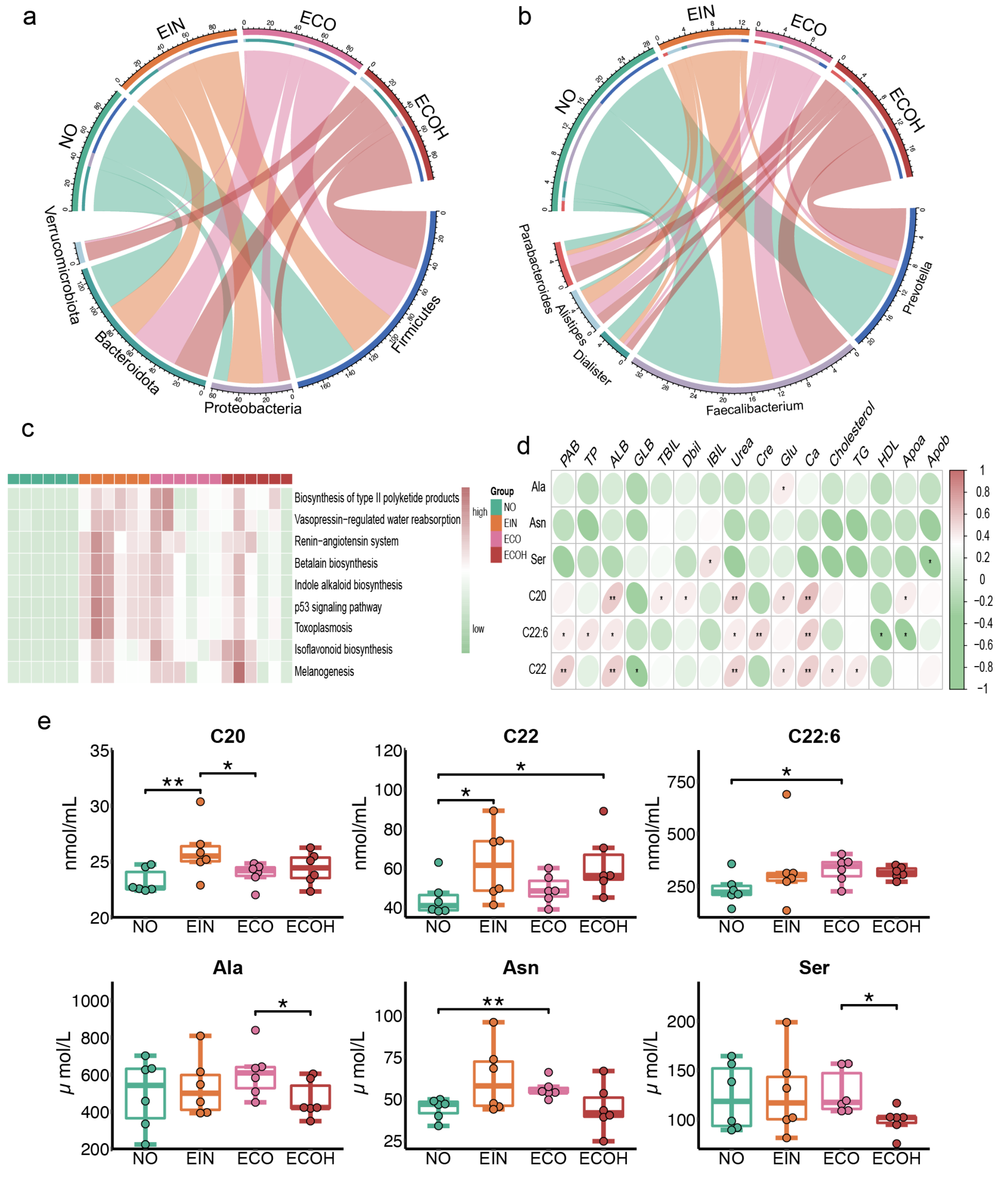

Supplementary figure S4. Shift of gut microbiota composition and metabolic characteristic in overweight patients. (a and b) Gut microbiota proportion in four groups at phylum and genus levels, respectively. (c) Alteration of predicted function of gut microbiome among NO, EIN, ECO and ECOH individuals. (d) Correlation analysis of

clinical indices and altered metabolites (Two-tailed Wilcox test, * $p < 0.05$, ** $p < 0.01$).
(e) Significantly changed circulating metabolites among overweight subjects.

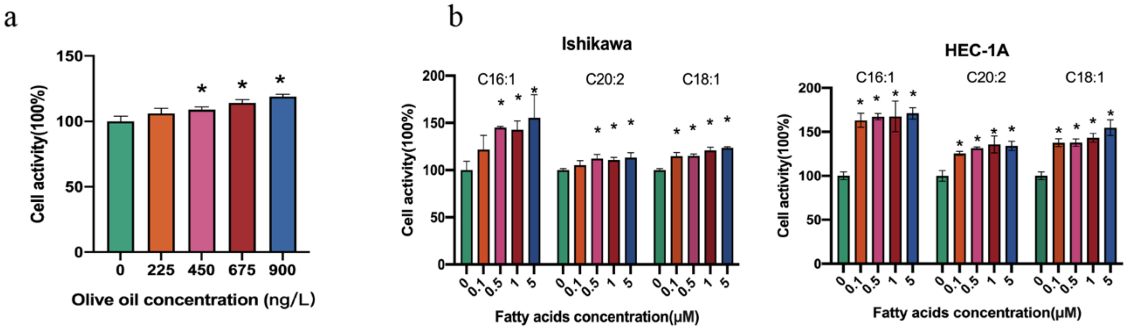

Supplementary figure S5. Fatty acids promoted EC proliferation in vitro. (a) Cell viability of Ishikawa cells pretreated by olive oil with various concentrations for 24 h that was analyzed by CCK-8 assay, compared with the control group (*p<0.05). (b) Cell viability of EC cells pretreated by C16:1, C20:2, C18:1 with various concentrations for 24 h that was analyzed by CCK-8 assay, Data compared with the control group (*p<0.05).

Table

Supplementary Table 1 Characteristics of study participants

Characteristic	EC (n=18)	Control (n=18)
Age, year(mean±SD)	54.94±9.7	47.47±6.88
Body-mass index(mean±SD)	28.05±5.31	25.24±3.97
Normal (BMI<25)	6	6
Overweight (BMI≥25)	12	12
Histologic subtype		
Endometrioid	15	-

Serous	2	-
Carcinosarcoma	1	-
Hysteromyoma	-	2
Adenomyosis	-	5
CIN	-	4
Endometrial polyp	-	1
EIN	-	6
FIGO stage		
IA	9	-
IB	3	-
II	1	-
III	5	-
IIIA	2	-
IIIB	0	-
IIIC1	0	-
IIIC2	3	-
IV	0	-
Diabetes (%)		
Yes	5 (27.8%)	1 (5.6%)
No	13(72.2%)	17(94.4%)
Hypertension (%)		
Yes	6 (33.3%)	1 (5.6%)
No	12(66.7%)	17(94.4%)
Postmenopausal (%)		
Yes	10(55.6%)	3 (16.7%)
No	8 (44.4%)	15(83.3%)
