# Supplementary material for: Altered Gut Microbial Profile Accompanied by Abnormal Fatty Acid Metabolism Activity Exacerbates Endometrial Cancer Progression
Source: Microbiol Spectr. 2022 Oct 13;10(6):e02612-22. doi: 10.1128/spectrum.02612-22 (PMC9769730; doi:10.1128/spectrum.02612-22)
Supplement: Supplemental file 2 — Table S1. Download spectrum.02612-22-s0002.pdf, PDF file, 0.1 MB [file spectrum.02612-22-s0002.pdf]

Supplementary Table 1 Characteristics of study participants

Characteristic	EC (n=18)	Control (n=18)
Age, year(mean±SD)	54.94±9.7	47.47±6.88
Body-mass index(mean±SD)	28.05±5.31	25.24±3.97
Normal (BMI<25)	6	6
Overweight (BMI≥25)	12	12
Histologic subtype		
Endometrioid	15	-
Serous	2	-
Carcinosarcoma	1	-
Hysteromyoma	-	2
Adenomyosis	-	5
CIN	-	4
Endometrial polyp	-	1
EIN	-	6
FIGO stage		
IA	9	-
IB	3	-
II	1	-
III	5	-
IIIA	2	-
IIIB	0	-
IIIC1	0	-
IIIC2	3	-
IV	0	-
Diabetes (%)		
Yes	5 (27.8%)	1 (5.6%)
No	13(72.2%)	17(94.4%)
Hypertension (%)		
Yes	6 (33.3%)	1 (5.6%)

No	12(66.7%)	17(94.4%)
Postmenopausal (%)		
Yes	10(55.6%)	3 (16.7%)
No	8 (44.4%)	15(83.3%)
